# Patient centred care in diabetology: an Islamic perspective from Iran

**DOI:** 10.1186/2251-6581-12-18

**Published:** 2013-05-10

**Authors:** Bagher Larijani, Farzaneh Zahedi

**Affiliations:** 1 Medical Ethics and History of Medicine Research Centre, Tehran University of Medical Sciences, Tehran, Iran; 2 Endocrinology and Metabolism Research Centre, Endocrinology and Metabolism Research Institute, Tehran University of Medical Sciences, Tehran, Iran

**Keywords:** Patient centered care, Diabetology, Diabetes mellitus, Religious ethics, Islam, Iran

## Abstract

Patient-centred system of care is essential in managing many disorders such as diabetes mellitus. The cultural and religious context can influence the involvement of patients and their families in such a care. We intend to discuss patient-centred care in diabetology in view of Islam. For more clarification, we will take into consideration a few illustrative lines of argument in detail about situation in Iran. In conclusion, dynamic spirit of Islamic jurisprudence is reflected in its adaptability to change in medical practice. In recent decades, Iranian religious scholars have provided scientists in new fields of science and research with appropriate directions and guidelines. Decree issued by Iranian religious leaders permitting research on stem cells for therapeutic purposes in many disorders including diabetes mellitus is one example. Understanding of the nature of Islam is importance for communication with patients in Islamic countries.

## Introduction

In comparison to provider centered care, in which health care providers follow determined goals by clinicians to manage acute and chronic diseases; patient centered care (PCC) looks for “*providing care that is respectful of and responsive to individual patient preferences, needs, and values and ensuring that patient values guide all clinical decisions*[1]”. It is evident that in a chronic disease like diabetes mellitus which needs close involvement of the patients and their families, traditional provider centered care is rarely beneficial. Patient centered system of care provides patients with the integrated culturally sensitive services. In religious people, medical team should understand and respond effectively to religious beliefs and interests.

Considering great interests to put patient centered care to use in diabetic clinics, we intend to discuss it in view of Islam. Islamic perspective on patient-centred care in diabetology has been addressed by *Niazi* and *Karla* from South Asia in a recently published article in this journal [2]. The authors rightfully conclude that “*…Islam supports PCC. Islam can be used a motivating factor for optimal health care*”. However, it seems that the authors focus only on India and Pakistan and the related sociocultural background; so, their points about Islamic environment cannot considered generalized and extended to all Islamic societies. Although we do not aim to address all the issues emerged by this article, we will discuss certain themes. For more clarification, we will take into consideration a few illustrative lines of argument in detail about situation in Iran.

## Considerations in Iran

Ever-increasingly, Patient-centered care has received a great attention in Iran in various fields including diabetes mellitus. Effectiveness of community-based and patient-centered care on glycemic control, patient satisfaction and quality of life of Iranian patients was confirmed in a clinical trial by Iranian Diabetes Association in 2003 [3]. National Diabetes Prevention and Control Program has adopted as a systematic approach for delivery of health care to diabetic patients all over the country [4,5]. In addition, Deputy for Research and the National Advisory Committee on Non-communicable Diseases of Ministry of Health endorsed the Iranian National Diabetes Research Network (INDIRAN) Project as the infrastructure for research in diabetes in 2002 [6]. Meanwhile, sub-specialty clinics with multidisciplinary teams have been established in big cities. As a case in point, Diabetes and Metabolic Disorders Clinic [7], established by Endocrinology and Metabolism Research Institute (EMRI) of Tehran University of Medical Sciences (TUMS) in 2009, provides diabetic patients with specialized medical services, and intends to reform diabetic care to a patient-centered one. Patient education is a main part of services. All patient and their close family learn about nature of the disease, how to control it and prevent complications. In such an environment, myths of “*disease process is related to supernatural entities*” or “*fighting against disease is in opposition to God’s will*” are far from being acceptable to Muslim patients in our country. As a popular belief in Iran which is rooted in the Islamic teachings, there is natural rationale behind all diseases. Islam encourages people to find out the causes of diseases and discover the other laws of the universe and use them for the benefit of mankind.

Whereas the article by *Niazi* and *Karla* which states “*The traditional Islamic religious leaders may not have exposure to current scientific education, and may at times be involved in the unintentional spread of harmful medical knowledge among the society….*”, religious leaders in Iran have played a significant role in paving the way for scientific promotion in the country in recent decades. Actually, dynamic spirit of Islamic jurisprudence in Iran is reflected in its adaptability to change in medical practice. Religious decrees (fatwa) issued by Muslim scholars guides diabetic patients in various fields from Insulin injection, using different medicines, fasting in Ramadan, and even in novel treatments such as therapeutic use of stem cells. Despite the opposing viewpoints on stem cell research in some countries, Iranian religious leaders issued decrees permitting research on stem cells for therapeutic purposes [8]. Accordingly, “*experimental activities and therapeutic uses of stem cells are permissible before ensoulment with necessary precautions when they are justifiable based on Islamic principles such as the public interest*” [9]. Looking for scientific advancements and seeking new treatments for human disorders may also apply to justify the use of human embryonic stem cells [8-10]. Owing to religious support of stem cell research [8], experiments for therapeutic use of stem cells in various disorders including diabetes mellitus have been carried out in Iran [11,12]. Cooperation between religious scholars and scientists has also paved the way for approval of some laws and ethical guidelines in the country [13-16].

The other point is that we cannot certainly agree with the details under the title of “*common myths and challenges in the Islamic society*”. It would be better to separate cultural traditions from religious beliefs and not refer the myths to Islamic teachings. Essentially, referring to relevant literature, particularly when we seek “Evidence from Hadiths” is needed. In other words, the supposed myths may be the cultural characteristics of a society or a kind of misconception. Actually, the realities are completely different in Iran, as another Islamic society. As a case in point, women in urban and rural areas in Iran are active in different fields and have normal outdoor activities. Interestingly, the ratio of female (to male) students in universities has a growing trend. Based on a study in 2007, the distribution of genders in higher education organizations was 48.87% male and 51.12% female [17]. The female ratio is close to 60% now. All things together, we think that many challenges and myths discussed by *Niazi* and *Karla* are related to the culture and social context, not to religious and Islamic views.

## Conclusion

To sum up, understanding of the nature of Islam is importance for communication with patients in Islamic countries or in cosmopolitan areas around the world. There are some misconceptions about Islamic beliefs of which many are related to sociocultural background of the societies. As mentioned, the challenges and myths pointed out by *Niazi* and *Karla* are mostly culturally bonded. However, we agree with the authors’ conclusion that reflects the positive position of Islam on patient centered care. Obviously, an efficient patient centered care, through a proactive approach, would respect both cultural and religious beliefs and respond the patients’ needs and preferences in the care delivery setting.

## Competing interests

The authors declare that they have no competing interests.

## Authors’ contributions

FZ wrote the first draft and final version of the article. BL edited the article. Both authors approved the final manuscript.
